# Novel multimodal MRI and MicroCT imaging approach to quantify angiogenesis and 3D vascular architecture of biomaterials

**DOI:** 10.1038/s41598-019-55411-4

**Published:** 2019-12-19

**Authors:** Anna Woloszyk, Petra Wolint, Anton S. Becker, Andreas Boss, Weston Fath, Yinghua Tian, Simon P. Hoerstrup, Johanna Buschmann, Maximilian Y. Emmert

**Affiliations:** 1 Division of Surgical Research, University Hospital of Zurich, Zurich, Switzerland; 2 Institute for Regenerative Medicine, University of Zurich, Zurich, Switzerland; 3 Department of Orthopaedics, University of Texas Health Science Center San Antonio, San Antonio, TX USA; 4 Institute for Diagnostic and Interventional Radiology, University Hospital of Zurich, Zurich, Switzerland; 5 Visceral and Transplant Surgery, University Hospital Zurich, Zurich, Switzerland; 6 Wyss Translational Center Zurich, University of Zurich & ETH Zurich, Zurich, Switzerland; 7 Clinic for Plastic Surgery and Hand Surgery, University Hospital Zurich, Zurich, Switzerland; 8 Department of Cardiovascular Surgery, Charité Universitätsmedizin Berlin, Berlin, Germany; 9 Department of Cardiothoracic and Vascular Surgery, German Heart Center Berlin, Berlin, Germany

**Keywords:** 3-D reconstruction, Magnetic resonance imaging, Angiogenesis

## Abstract

Quantitative assessment of functional perfusion capacity and vessel architecture is critical when validating biomaterials for regenerative medicine purposes and requires high-tech analytical methods. Here, combining two clinically relevant imaging techniques, (magnetic resonance imaging; MRI and microcomputed tomography; MicroCT) and using the chorioallantoic membrane (CAM) assay, we present and validate a novel functional and morphological three-dimensional (3D) analysis strategy to study neovascularization in biomaterials relevant for bone regeneration. Using our new pump-assisted approach, the two scaffolds, Optimaix (laminar structure mimicking entities of the diaphysis) and DegraPol (highly porous resembling spongy bone), were shown to directly affect the architecture of the ingrowing neovasculature. Perfusion capacity (MRI) and total vessel volume (MicroCT) strongly correlated for both biomaterials, suggesting that our approach allows for a comprehensive evaluation of the vascularization pattern and efficiency of biomaterials. Being compliant with the 3R-principles (replacement, reduction and refinement), the well-established and easy-to-handle CAM model offers many advantages such as low costs, immune-incompetence and short experimental times with high-grade read-outs when compared to conventional animal models. Therefore, combined with our imaging-guided approach it represents a powerful tool to study angiogenesis in biomaterials.

## Introduction

Tissue engineering often utilizes suitable biomaterials intended to guide and stimulate the healing process of the body^1–4^. Most tissues are highly vascularized, such as skin^5^, bone^6^ or the cardiac muscle^7^. New therapies using tissue engineering aim to improve the limited regenerative potential of native tissue by restoring the function and morphology of the damaged tissue or organ. Therefore, implantable biomaterials play an essential role and must have a strong vascularization capacity, which enables efficient integration at the site of implantation thereby promoting repair and regeneration. Microporous materials provide a 3D architecture and stimulate vessel ingrowth from the surrounding tissues. Imaging the vascularization of tissue-engineered constructs (TECs), therefore, is a crucial step in establishing a functional and adequately vascularized TEC-based implant.

To assess the vascularization potential of a biomaterial, numerous imaging and quantification techniques are available. *Ex vivo* analysis can either be performed using histological sections in combination with immunolabeling of endothelial cell markers^8,9^, corrosion casts^10,11^, advanced microscopic techniques, such as e.g. confocal laser scanning microscopy^12^, or microcomputed tomography (MicroCT) combined with radiopaque contrast agents^13^. *In vivo* techniques include high-frequency ultrasound, optoacoustic imaging, two-photon microscopy and magnetic resonance imaging (MRI)^9,14–22^, and serve as stand-alone analysis methods or can be combined with corresponding *ex vivo* imaging techniques.

However, not all of these methods are precise or quantitative enough as they are only focused on a small area with a limited depth of field. In fact, histological assessment remains the most widely used technique to analyse blood vessels. It is however limited to two dimensions and it is not always possible to distinguish between functional and non-functional blood vessels^23^. Finally, histological analysis makes the spatial distribution of vascular elements and differences on the cellular level between different blood vessels visible, but the vascular architecture and thus the vascular network cannot be represented in a 3D format.

Several studies have used a combination of the non-destructive and non-invasive *in vivo* MRI followed by *ex vivo* MicroCT analysis after perfusion of the sample with radiopaque contrast agents for imaging vascularization in biomaterials^24^, bone TECs^25^, and tumours^26,27^. Ribot *et al*. used MRI and MicroCT for the longitudinal evaluation of neo-vascularization in biomaterials that were implanted in a femoral defect in rats. MRI results in this study were confirmed by MicroCT and histology, which highlighted the efficacy of longitudinal MRI for tissue engineering^24^. However, most of the studies using MRI and MicroCT for vascular imaging and quantification were performed in large (e.g. sheep^25^) and small (e.g. rabbit^27^ and rats^24^) animal models. Unfortunately, their application is linked to high costs, animal burden and often very time-consuming experiments. In addition, immunological reactions are induced if xenogenic cells are required and lead to the necessity of immunocompromised models.

Recently, the chorioallantoic membrane (CAM) assay of the living chicken embryo has gained considerable attention as it offers a cost- and time-effective, less sentient as well as easily manageable alternative to other common animal models. The use of the CAM as an *in vivo* bioreactor allows for testing the biocompatibility of biomaterials^28^, assessing bone repair in injured bone cylinders from human femoral heads placed on the CAM by MicroCT^29^, or studying *in vivo* functional perfusion capacity of TECs by MRI^9,14^ while keeping cells and organs alive.

In this study, we present a novel, multimodal approach to comprehensively assess functional and morphological neoangiogenesis using the CAM model. To validate our novel approach, two biomaterials highly relevant for bone regeneration, but structurally different, were used including a natural biomaterial produced from porcine collagen (Optimaix)^30^, and a synthetic polymer scaffold (DegraPol)^31^. The main hypotheses were:

(i) whether and to what extent the perfusion capacity as assessed by MRI (functional neoangiogenesis) can be correlated with the total vessel density and volume as measured by MicroCT (morphological neoangiogenesis) within the same scaffold; and
(ii) whether the two biomaterials show different vascularization patterns that can be visualized and quantified with MRI and MicroCT.

**Figure 1:**
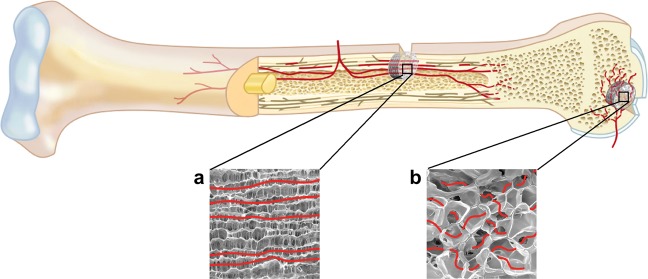
Potential application of Optimaix and DegraPol scaffolds in bone regeneration. The structure of the respective biomaterial guides ingrowing neovessels. The architecture of (**a**) the lamellar structure of the natural collagen scaffold (Optimaix) is appropriate for replacement in diaphyseal defects, while (**b**) the highly porous polymer foam (DegraPol) is predestined for replacement in epiphysis defects.

To test this, both biomaterials were incubated on the same CAM for one week and a combined functional and morphological evaluation of neoangiogenesis using perfusion MRI, MicroCT and histological analyses were performed to assess their therapeutic potential for bone regeneration. Due to its architecture, the lamellar structure of the collagen scaffold might be suitable for replacements in diaphyseal defects, whereas the highly porous DegraPol foam resembles the vascular network in the spongy bone of the epiphysis (Fig. 1).

For functional MRI measurements, images of pre- and post-injection of a Gadolinium-based contrast agent were analysed for T1 relaxation times. MicroCT assessments were performed after blood vessel perfusion with MicroFil. A new semi-automatic perfusion technique was developed for even perfusion of the vessels. In addition, to characterize the vessel architecture, we also determined the vessel volume, the number of branches, junctions, branches per junctions, total and mean branch lengths as well as the mean radius based on quantitative MicroCT images. Finally, the scaffolds were histologically analysed and examined for vessel density, length and angle in a semi-automated procedure.

## Results

### Comprehensive MicroCT analysis: feasibility and validation

**Figure 2:**
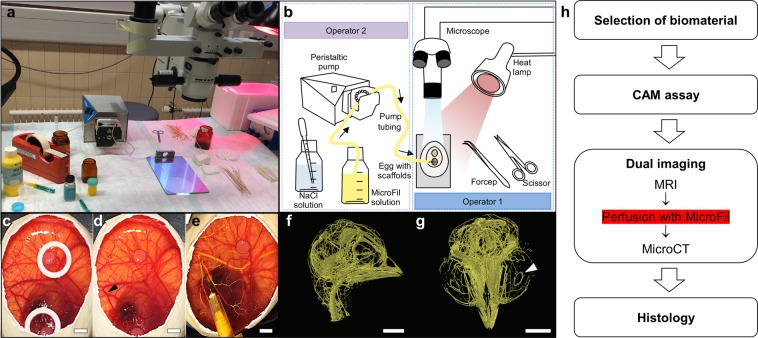
Setup for the pump-assisted CAM perfusion. (**a**) Photograph shows the setup of the workstation and equipment for the pump-assisted perfusion of the CAM with radiopaque MicroFil by two operators. (**b**) Detailed schematic drawing of the required equipment. Operator 2 operates the pump and sprinkles the CAM with NaCl solution to avoid dehydration during the perfusion process before Operator 1 places the needle of the MicroFil tube under microscopic view into a superficial blood vessel. (**c**) Scaffold (black dotted line) was incubated on the CAM for 7 days stabilized by a silicone ring. (**d**) The ring is removed for perfusion and a suitable vessel for the puncture site is selected (arrow). (**e**) The exchange of blood with the yellow MicroFil reagent indicates successful perfusion of the vessels. (**f**) MicroCT image of lateral view of the MicroFil-perfused chicken embryo head. (**g**) Coronal view of the MicroFil-perfused chicken embryo head. The white arrow indicates the major circulus arteriosus of the iris. We refer the reader to Supplementary Video S1. (**h**) Flowchart shows the whole process needed to evaluate a biomaterial for angiogenesis using the novel multimodal MRI and MicroCT imaging approach. The pump-assisted perfusion with MicroFil is performed after the MRI measurement. Scale bars = 500 μm.

To investigate the technical quality of the newly developed pump-supported MicroFil perfusion of the scaffolds (Fig. 2) and the following semi-automatic computer-based evaluation of the data collected by MicroCT.

**Figure 3:**
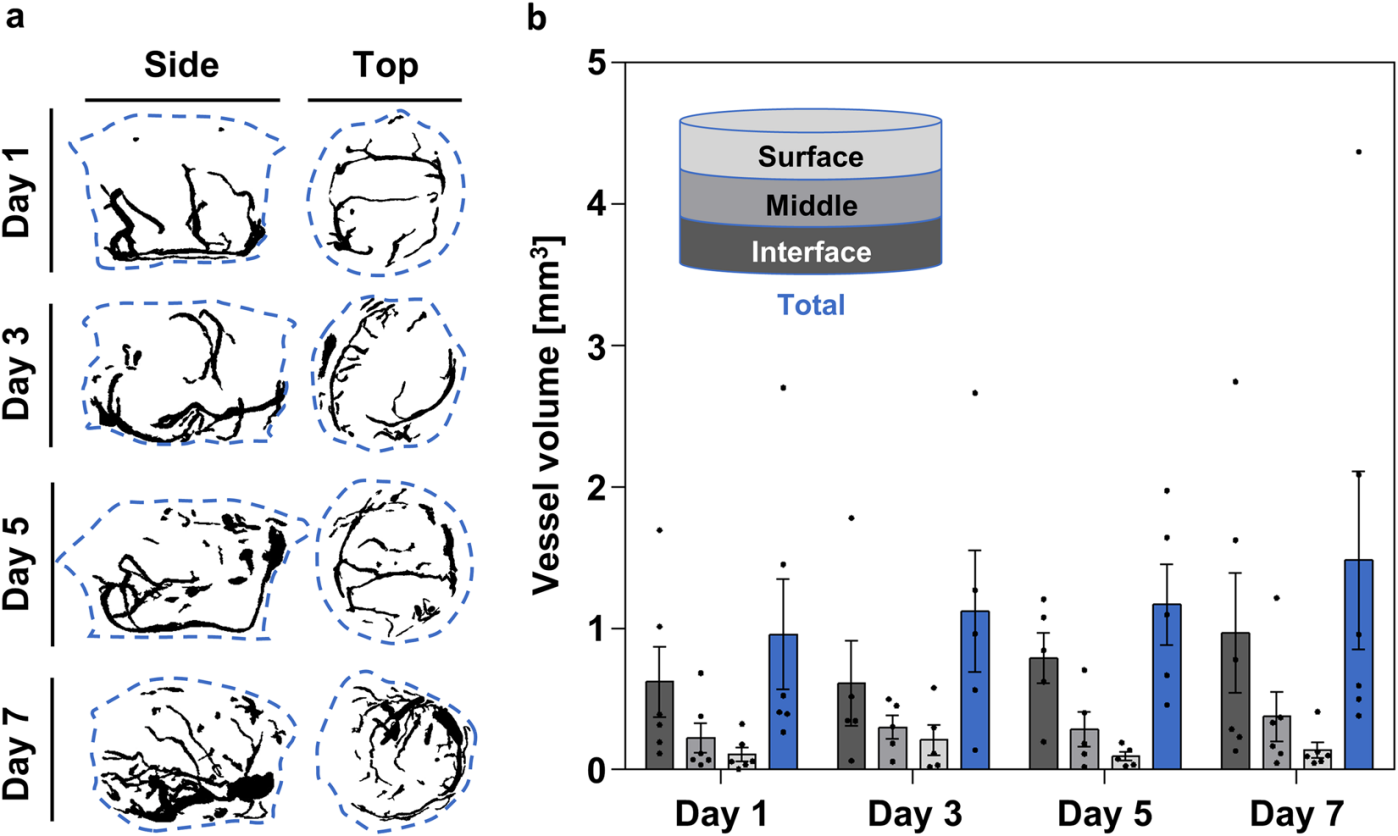
Validation of the vessel quantification method using the newly developed pump-assisted MicroFil perfusion and MicroCT scanning. (**a**) Representative maximum intensity projections of side and top views, day 1–7. (**b**) Vessel volume found in Optimaix scaffolds after 1, 3, 5, and 7 days of *in ovo* incubation. All measurements were taken from distinct samples. Mean ± SE, n = 5–6.

A time course experiment was performed in Optimaix to validate the vessel quantification method using MicroFil perfusion and MicroCT. The vessel volume was measured on days 1, 3, 5, and 7 in the interface, middle, and surface layers of each sample. The results showed the expected gradual increase in total vessel volume from day 1 to day 7, which increased from 0.96 ± 0.39 mm^3^ to 1.48 ± 0.63 mm^3^ (Mean ± SE) corresponding to a 54.17% increase in total vessel volume within the incubation period (Fig. 3a, blue bars in b). The separate analysis of the (CAM-)interface, middle, and surface regions of the scaffolds showed a vascularization gradient at all experimental time points, with more vascularization at the interface, which gradually decreased towards the surface (Fig. 3a,b). This was also evident in the vascular architecture, which had developed from a simple structure with a few branches (day 1) to a more complex network of vessels (day 7; Fig. 3a).

### Establishment of combined *in vivo* MRI and *ex vivo* MicroCT approach

**Figure 4:**
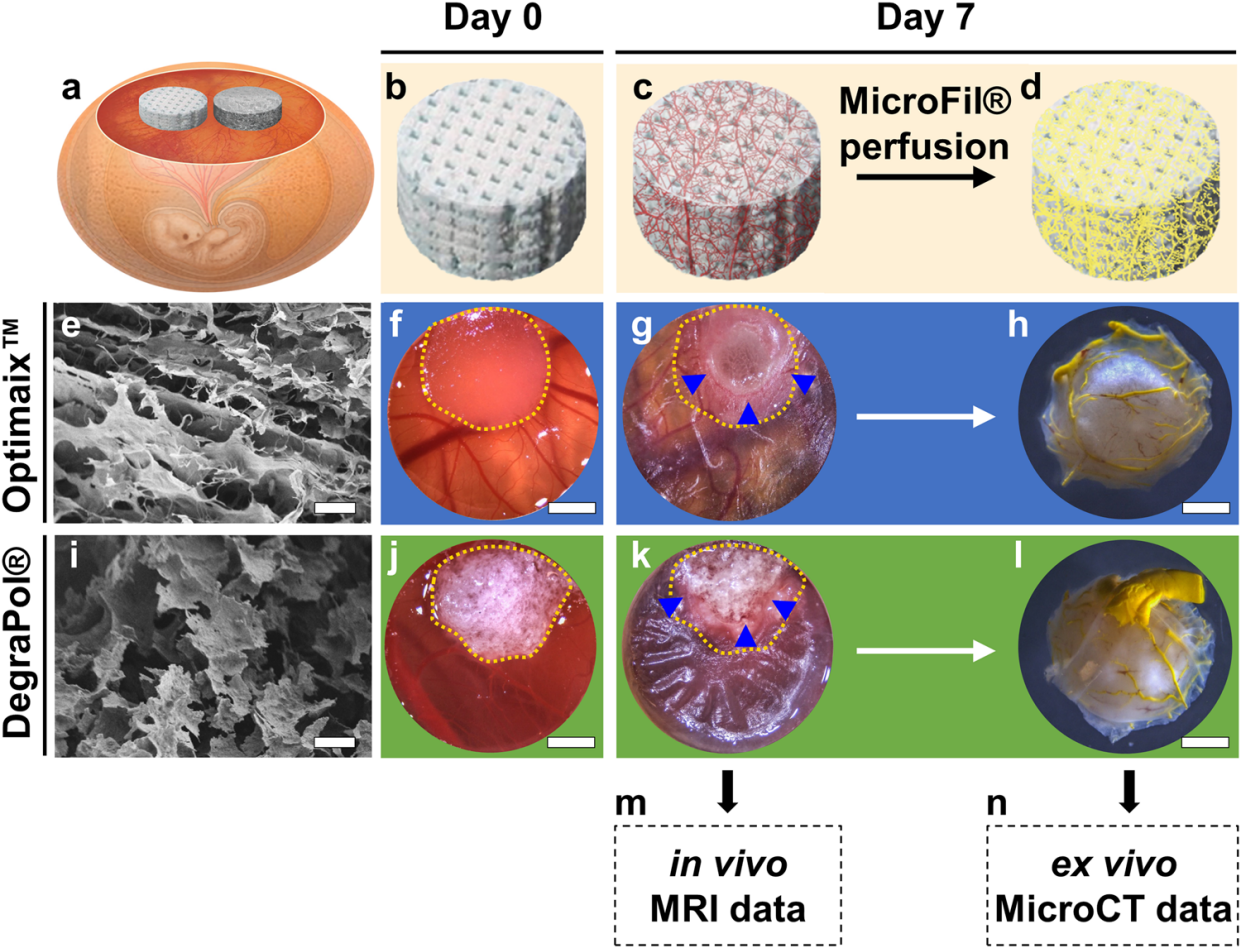
Experimental setup. (**a**) Chorioallantoic membrane (CAM) assay with two different biomaterials that are investigated in the same organism. (**b**) Empty scaffold on day 0. (**c**) Vascularized scaffold on day 7 as used for (**m**) magnetic resonance imaging (MRI). (**d**) Scaffold after perfusion with yellow MicroFil as used for (**n**) microcomputed tomography (MicroCT). (**e**) Scanning electron microscopy (SEM) image showing the lamellar structure of the Optimaix scaffold at 100x magnification. Scale bar = 100 μm. (**f**) Optimaix scaffold right after placement on the CAM and (**g**) after 7 days of incubation on the CAM. (**h**) Bottom view of the same sample after perfusion with MicroFil. (**i**) SEM of the DegraPol scaffold at 100x magnification shows a highly porous foam structure. Scale bar = 100 μm. (**j**) DegraPol scaffold right after placement on the CAM and (**k**) after 7 days of incubation on the CAM. (**l**) Bottom view of the same sample after perfusion with MicroFil. The yellow dotted lines in (**f**,**g**,**j**,**k)** indicate the outline of the scaffolds on day 0. Blue arrowheads point at capillaries at the edge between CAM and scaffold. Scale bars in (**f–h,j–l**) = 200 μm.

After 7 days of *in ovo* incubation (Fig. 4a), the initially empty microporous scaffolds (Fig. 4b,e,i) contained a vascular network growing in from the CAM (Fig. 4c). MRI scans (Fig. 4m) were performed immediately before perfusing the vasculature with MicroFil (Fig. 4d) for visualization with MicroCT (Fig. 4n).

Macroscopic evaluation of both Optimaix (Fig. 4e) and DegraPol (Fig. 4i) biomaterials placed on the CAM (Fig. 4f,j) did not reveal any apparent differences between the two biomaterials regarding the interaction of the CAM with the material. In both groups the highly vascularized CAM gradually engulfed the scaffolds (Fig. 4f,g,j,k, yellow dotted line shows the original scaffold size on day 0) and blood capillaries were visible around the scaffolds at the end of the day 7 *in ovo* incubation period (Fig. 4g,k, blue arrowheads). Macroscopic images of the MicroFil-perfused scaffolds showed successful replacement of the blood with MicroFil in both biomaterials (Fig. 4h,l).

### Correlation of MRI and MicroCT data

**Figure 5:**
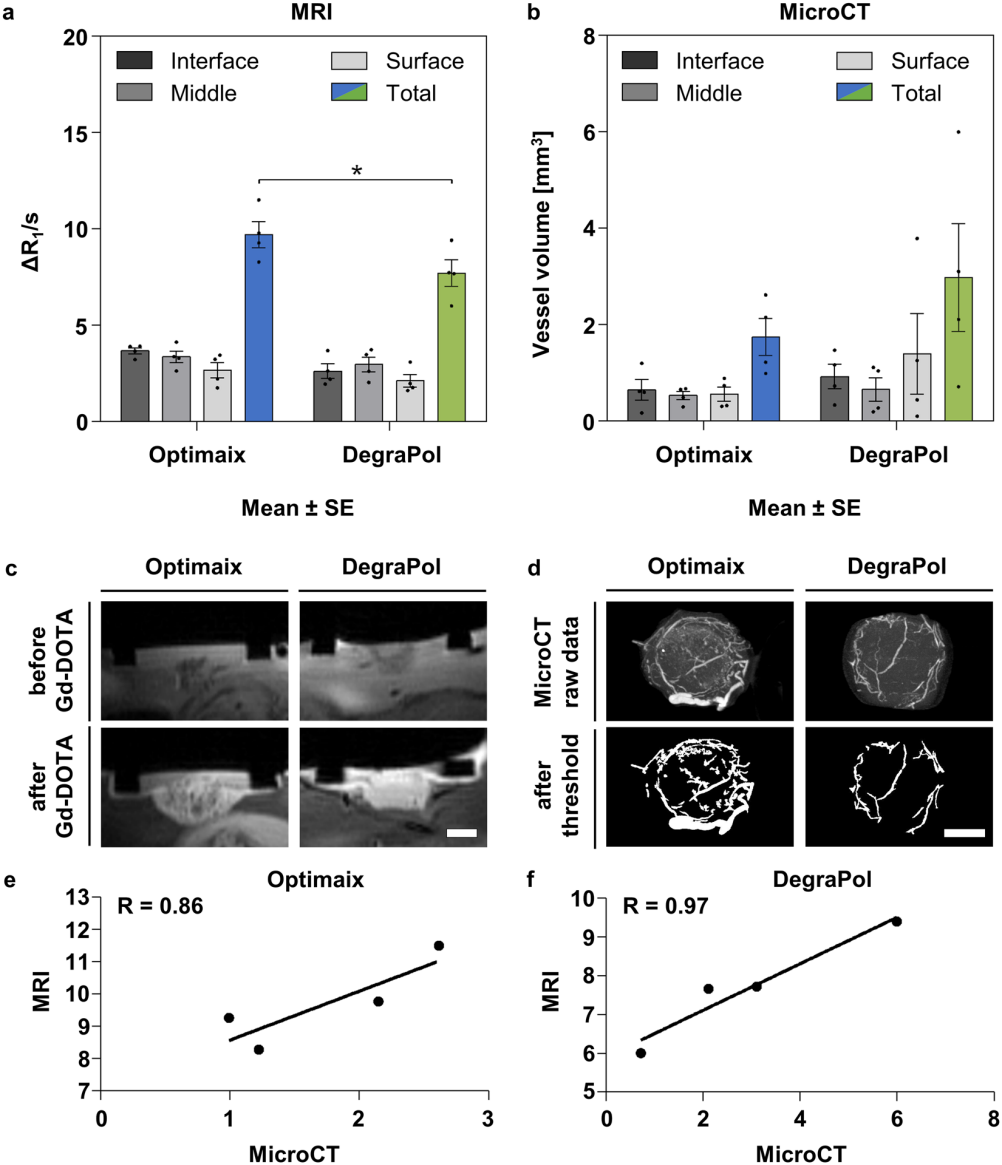
Imaging and analysis of the vasculature within Optimaix and DegraPol scaffolds. (**a**) Functional assessment of blood perfusion capacity by MRI. Mean ± SE, n = 4, *p < 0.05. (**b**) Vessel volume determined by MicroCT after MicroFil perfusion of the vasculature. Mean ± SE, n = 4. (**c**) Representative magnetic resonance image of Optimaix and DegraPol scaffolds on the CAM before and after injecting paramagnetic Gadolinium-DOTA contrast agent. (**d**) Representative maximum intensity projections of MicroCT scans and of binarized images for both Optimaix and DegraPol scaffolds. (**e**) Correlation of total blood perfusion capacity (MRI) and total vessel volume (MicroCT) for Optimaix scaffolds. (**f**) Correlation of total blood perfusion capacity (MRI) and total vessel volume (MicroCT) for DegraPol scaffolds. Scale bars = 250 μm.

The MRI data showed no significant difference between the three layers in either of the two scaffolds, however, a gradual decrease in perfusion capacity from the interface to the middle and surface layers of 9% and 27%, respectively, was observed in the Optimaix scaffolds. In DegraPol scaffolds the mean blood perfusion in the middle layer was higher than in the interface layer by 13%, whereas the perfusion capacity within the surface layer was 19% lower compared to the interface. In total, the Optimaix group showed a 26% higher perfusion capacity relative to the DegraPol group (Fig. 5a,c).

Optimaix scaffolds were found to have a very homogeneous distribution of vasculature throughout the scaffolds showing only a small decrease in vessel volume in both the middle and surface layers by 18% and 14% when compared to the interface layer, respectively (Fig. 5b). In DegraPol scaffolds the largest vessel volume was found in the middle layer, followed by interface (−12%) and surface (−29%). In contrast to the MRI results, the MicroCT data revealed more vascularization in DegraPol scaffolds, showing a 1.7-fold increase in total vessel volume compared to Optimaix (Fig. 5b,d). The correlation coefficient R between MRI (total perfusion capacity) and MicroCT (total vessel volume) data for both Optimaix and DegraPol scaffolds was 0.86 and 0.97, respectively (Fig. 5e,f), confirming the suitability of combining MRI and MicroCT in this novel quantitative assessment of angiogenesis and vascular architecture.

### Quantitative analysis of the blood vessel architecture using MicroCT

**Figure 6:**
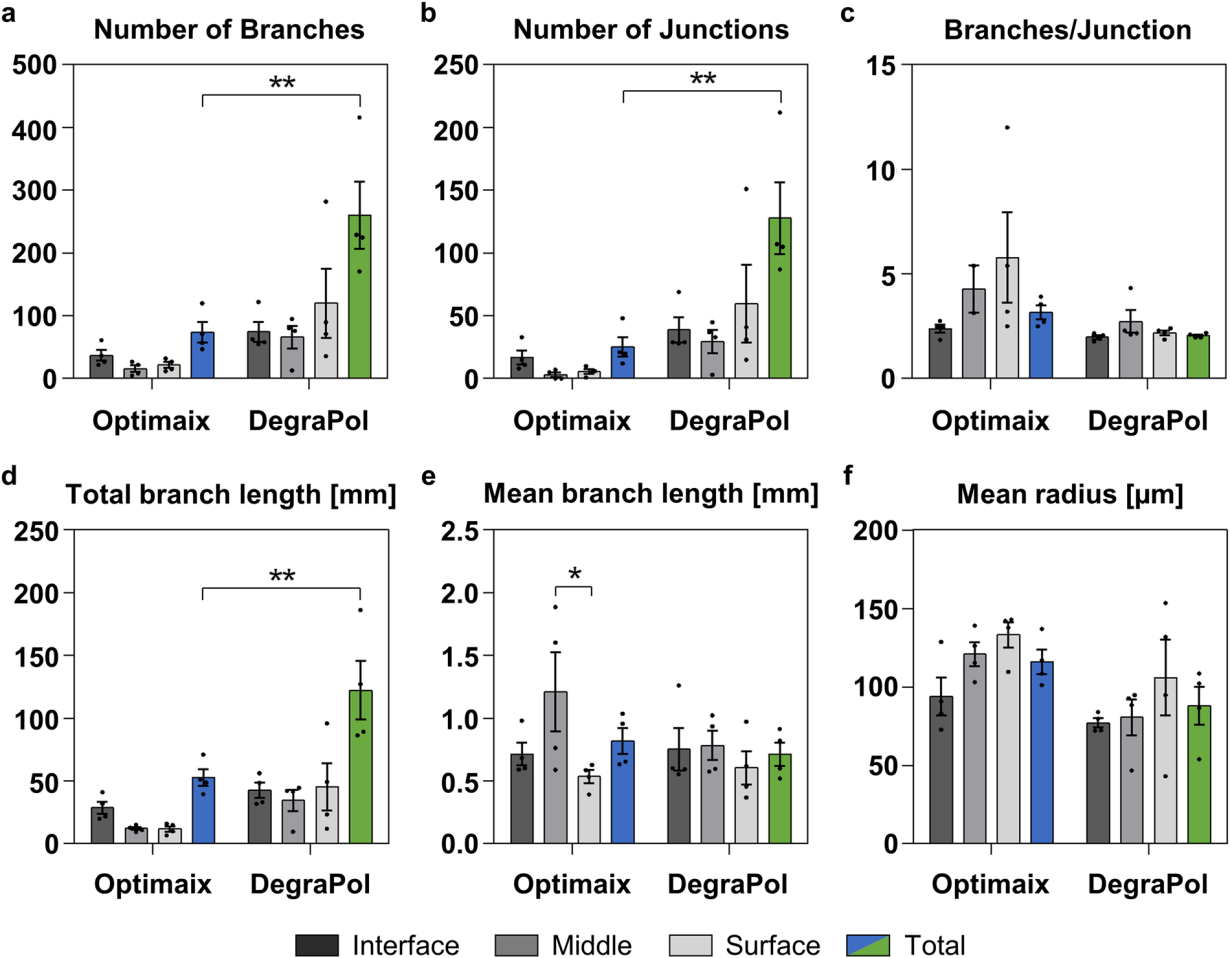
Vessel architecture in MicroFil-perfused Optimaix and DegraPol scaffolds. (**a**) Number of branches. (**b**) Number of junctions. (**c**) Number of branches per junction. (**d**) Total branch length in mm. (**e**) Mean branch length in mm. (**f**) Mean radius in μm. Mean ± SE, n = 4, *p < 0.05, **p < 0.01.

DegraPol demonstrated a higher number of branches (3.5-fold) and junctions (5.1-fold), when comparing the values measured in the total volume of the two scaffold types (Fig. 6a,b). The number of branches per junction increased from the interface towards the surface layer in Optimaix scaffolds, while it remained similar within the layers of the DegraPol scaffolds (Fig. 6c). As previously observed for the number of branches (Fig. 6a), the total branch length was more homogeneously distributed in DegraPol scaffolds and 2.3-fold higher than in Optimaix scaffolds, which contained half of the total branch length in the interface, while the middle and surface equally shared the other half (Fig. 6d). The mean branch length was similar in both scaffold types with very similar values for all three layers of the DegraPol scaffolds. In contrast, the Optimaix scaffolds on average had the longest branches in the middle layer, while the interface’s mean branch length was similar to what was calculated for the entire scaffold, and the surface had the shortest branches on average (Fig. 6e). The mean radius was a bit higher in Optimaix than in DegraPol scaffolds (1.3-fold). Interestingly, both scaffold types showed a gradual increase in mean radius from interface to surface, suggesting a material-independent architectural characteristic of the ingrowing neovessels (Fig. 6f).

### Influence of microcalcification on MicroCT data processing

**Figure 7:**
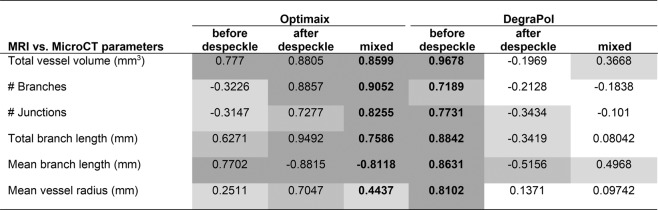
Correlation coefficients R for the comparison of MicroCT readouts with perfusion capacity assessed by MRI for the whole scaffolds (total). Correlation coefficients for the three layers can be found in Supplementary Table S1. Key: mixed = interface without despeckling and middle and surface with despeckling; white −0.25 < R < 0.25 (no correlation); light grey −0.5 < R < −0.25 & 0.25 < R < 0.5 (weak correlation); medium grey −0.75 < R < −0.5 & 0.5 < R < 0.75 (moderate correlation); dark grey −1 < R < −0.75 & 0.75 < R < 1 (strong correlation); bold font shows the chosen analysis setup for each scaffold.

In the Optimaix scaffold, an increase in microcalcification on the surface of the biomaterial was observed over time. While the mineralization did not influence the MRI measurements, the MicroCT detected the spots with the same intensity as the blood vessels perfused with contrast agent. This led to false positive vessels assessed by the software, that were unrelated to the perfused vasculature. For this reason, it was necessary to perform a despeckling step in the post-processing for the middle and surface layers (Fig. 7, ‘Optimaix mixed’). For DegraPol, no despeckling step was needed, because this synthetic scaffold did not evoke microcalcifications. Even if such a despeckling step was also applied to DegraPol scaffolds, it did not influence the final relative comparison of vessel architecture of the two biomaterials.

As such, relative relaxation rates (MRI) and total vessel volume (MicroCT) correlated well for both biomaterials. Even parameters that are considered less important than the total vessel volume correlated well with relative relaxation rates, such as branches or junctions. Impressively, for Optimaix, with the exception of mean radius, all parameters assessed by MicroCT correlated with R > 0.76 (Fig. 7, ‘Optimaix mixed’), while all parameters measured in DegraPol showed a correlation of more than 0.72 (Fig. 7, ‘DegraPol before despeckle’). Overall these results show that the two methods correlate well, justifying the combined assessment of both functional and morphological properties of vascularization (Fig. 7, Supplementary Table S1).

### Histological analysis

Histomorphometry was performed on histological sections for comparison with the newly combined analysis of MRI and MicroCT. Even though the results cannot be compared directly, the branch density (Supplementary Fig. S1), which is comparable to the number of branches (Fig. 6a) demonstrated a similar trend in both analyses with DegraPol scaffolds showing a higher number of branches than Optimaix. Furthermore, the total branch length (compare Fig. 6d to Supplementary Fig. 1) and the mean branch length (compare Fig. 6e to Supplementary Fig. 1) correlated with the architectural parameters previously determined using the MicroCT data. In contrast, the mean radius measured in the histological sections showed an opposite trend when compared to the MicroCT data. However, the radius was derived from the minor value given by ImageJ’s Particle Analysis, which is the secondary axis of the best fitting ellipse fitted into each prelabelled vessel. Therefore, it might not be accurate enough to represent the actual radius and should rather be considered as a rough estimation. The percent vessel area was found to be very similar in both scaffold groups, whereas the angle difference to 90° was shown to be higher in DegraPol scaffolds, as expected. A higher value indicates that the direction of the vessels is less perpendicular to the surface of the CAM. The lower average value in Optimaix scaffolds shows that the vessels were growing along the tube-like pores of the Optimaix scaffold (Supplementary Fig. 1). Good correlation was found for MRI data and the total vessel volume (MicroCT) when compared to the Mean branch length (histology) and the Vessel area (histology) in both scaffold groups. Furthermore, in Optimaix scaffolds, good correlation was observed for the Mean branch length and the Mean radius determined by MicroCT and histology (data not shown). Nevertheless, the histological analysis remains a two-dimensional (2D) analysis that can never consider the entire sample size as only a limited number of sections can be analysed. Instead of absolute values only relative values can be obtained.

## Discussion

Imaging the function and structure of vessels in tissue engineered constructs (TECs) is essential to predict and validate their potential *in vivo* applicability and performance^24,32–34^. To achieve this accurately, in-depth visualization and quantification of the vascular system is mandatory. This includes i) the evaluation of vessel functionality as assessed by blood volume circulating within the vascular system, as well as ii) the analysis of vascular structure and morphology comprising the total amount of vessels, vessel length, number of branches, number of junctions, the total and mean branch length, and the mean radius in the different regions of the TE implant^35^. The combination of both functional and morphological analyses is important, especially since immature neo-vessels are often not instantly perfused, and thus not fully functional from the beginning, which is crucial to ensure adequate oxygen and nutrient supply of the corresponding tissue.

### The CAM assay as an efficient model system to assess functional and structural 3D angiogenesis in biomaterials for regenerative medicine

In this study we present and validate a novel approach using the CAM *in ovo* model system to comprehensively assess functional and morphological neoangiogenesis in biomaterials by combining two clinically relevant imaging techniques (MRI and MicroCT) that were separately adapted for *in ovo* use in our previous studies^9,36^. After two scaffolds designed for bone regeneration were placed and incubated on top of the CAM for one week, we evaluated the perfusion capacity by MRI *in vivo* and the micro-angioarchitecture by high-resolution MicroCT *ex vivo* within the same biomaterial in order to evaluate if and to what extent the functional neoangiogenesis can be correlated to the morphological neoangiogenesis.

Our results demonstrated high correlation coefficients for both biomaterials (Optimaix 0.86 and DegraPol 0.97) indicating that the perfusion capacity (MRI) and total vessel volume (MicroCT) do strongly correlate in the evaluated biomaterials. This is an important finding showing for the first time the principal feasibility to use the CAM *in ovo* model system to collect and combine functional and morphological 3D neovascularization data in biomaterials using a novel multimodal imaging approach. So far, most studies analysed the vascularization of biomaterials cultured on the CAM in 2D either by counting the number of vessels that invaded the biomaterial and/or through a histomorphometrical evaluation^29,37,38^. Therefore, moving from 2D to 3D analyses of vascularized biomaterials cultured on the CAM is an important step to help replacing, reducing, and refining (3 R rule)^39^ the use of rodents for the sole purpose of pre-screening large numbers of clinically relevant biomaterials.

### Assessment of biomaterial specific vascularization patterns

Next, we performed a comparative analysis between the two biomaterials and tested the hypothesis whether the two biomaterials display different vascularization patterns that can be visualized and quantified with MRI and MicroCT. Interestingly, while the correlation coefficient was slightly lower for the Optimaix scaffold, it demonstrated a higher degree of vascularization (26% higher perfusion capacity) when compared to the DegraPol scaffold. Furthermore, detailed analysis of branches, junctions, vessel length and radius revealed fundamental differences between the two biomaterials regarding structure and architecture of the newly formed vascular network.

In this context, it is to be noted that the two post-processing methods of the MicroCT data used in our study, with despeckling for the middle and surface layer (mixed) in Optimaix scaffolds and without despeckling for the DegraPol scaffolds, had several consequences for the correlations of relative relaxation rates as assessed by MRI versus the morphological parameters analysed in MicroCT 3D stacks. In order to compare the vascularization patterns for the two biomaterials, we used MicroCT data for Optimaix after mixed despeckling and data for DegraPol without despeckling. It was found that the highly porous DegraPol exhibited significantly higher numbers of branches, number of junctions, and total branch length, however, lower number of branches per junction, mean branch length, and lower mean radius when compared to Optimaix. In fact, based on the different architectures of the two scaffolds such differences in vascularization patterns were anticipated. As the Optimaix scaffold has a lamellar structure with channels from the bottom to the top acting as guiding structures for vessels, neovascularization from the CAM interface upwards resulted in longer, thicker vessels with less branches as compared to the heterogeneously distributed pores within the DegraPol scaffolds. DegraPol rather supported extended branching with shorter vessels, having smaller diameters and cross-sections compared to Optimaix. The importance of a guiding structure on the healing outcome of a critical size femoral defect was recently demonstrated by Petersen *et al*., who used the channel-like pores of the Optimaix scaffold to induce directional endochondral ossification to improve bone repair in a rat model^30^. Consistent with our findings in the CAM assay, blood capillaries were observed to grow from the bone marrow towards the mineralization front guided by the scaffold’s structure.

The pore size of scaffold materials may have an impact on the number and functionality of vessels that grow into the scaffolds. A study by Druecke *et al*. demonstrated that vessel density, vessel diameter and red blood cell velocity increased with increasing pore size in poly(ether ester) block-copolymers with pore sizes of 20–75 µm, 75–212 µm or 250–300 µm^40^. They examined these effects in the dorsal skinfold chamber of mice (time frame: longitudinal up to 20 days). The DegraPol foam used here exhibits a pore size of 300–400 µm, which is even larger than the largest pore size studied by Druecke *et al*. Hence, a widespread (throughout the whole scaffold) and functional vascularization after 7 days on the CAM assay is not surprising.

A generally accepted pore size limit for a proper vascularization of tissue engineered bone constructs (rule of thumb) is 250 µm; in other words, pore sizes <250 µm lead to lower vascularization, while pore sizes >250 µm lead to homogenous, fast and well-vascularized constructs^41^. Depending on the tissue to be replaced, however, it may be different. For soft tissue engineering, smaller pore sizes may be favourable. It has been shown that in nanocellulose constructs with different pore sizes, smaller ones, such as 60 µm, were better in terms of cell and tissue infiltration *in vivo* as well as with regard to deposition of extracellular matrix than larger one^42^, which had been supported by a better attachment of fibroblasts in this nanocellulose material *in vitro*^43^. It has to be emphasized, however, that in this study neovascularization was only scored semi-quantitatively^42^, which does not allow a direct quantitative comparison.

In addition, the structure of the pores may have relevant impacts on vascularization. As shown for a beta-TCP scaffold, channels (tunnels) were more vascularized than random interconnected pores^44^. In addition to different pore sizes for Optimaix and DegraPol, respectively, we also had different structures, such as tunnels in Optimaix and randomly interconnected pores in DegraPol foam. This may in addition lead to the different vascularization patterns observed in this study.

What about nanoporous scaffolds? Nano-sized holes are usually too small to allow proper vessel ingrowth; however, nanoporous materials may be an alternative to microporous materials and nevertheless improve vascularization. For example, electrospun fiber meshes where the fibers had nanoholes allowed better and faster vascularization because of the inherent nanotopography of such fibers^45^. Likewise, endothelial cells cultured on nanoyarn formed more capillary-like structured than on electrospun nanofibers of the same material^46^. Again, nanopatterned materials may act beneficially because of topographical cues rather than acting via provision of space, which is typical for materials with larger pores of hundreds of µm like in Optimaix and DegraPol.

### Strengths and challenges of our novel approach and comparison to other *in vivo* models

**Figure 8:**
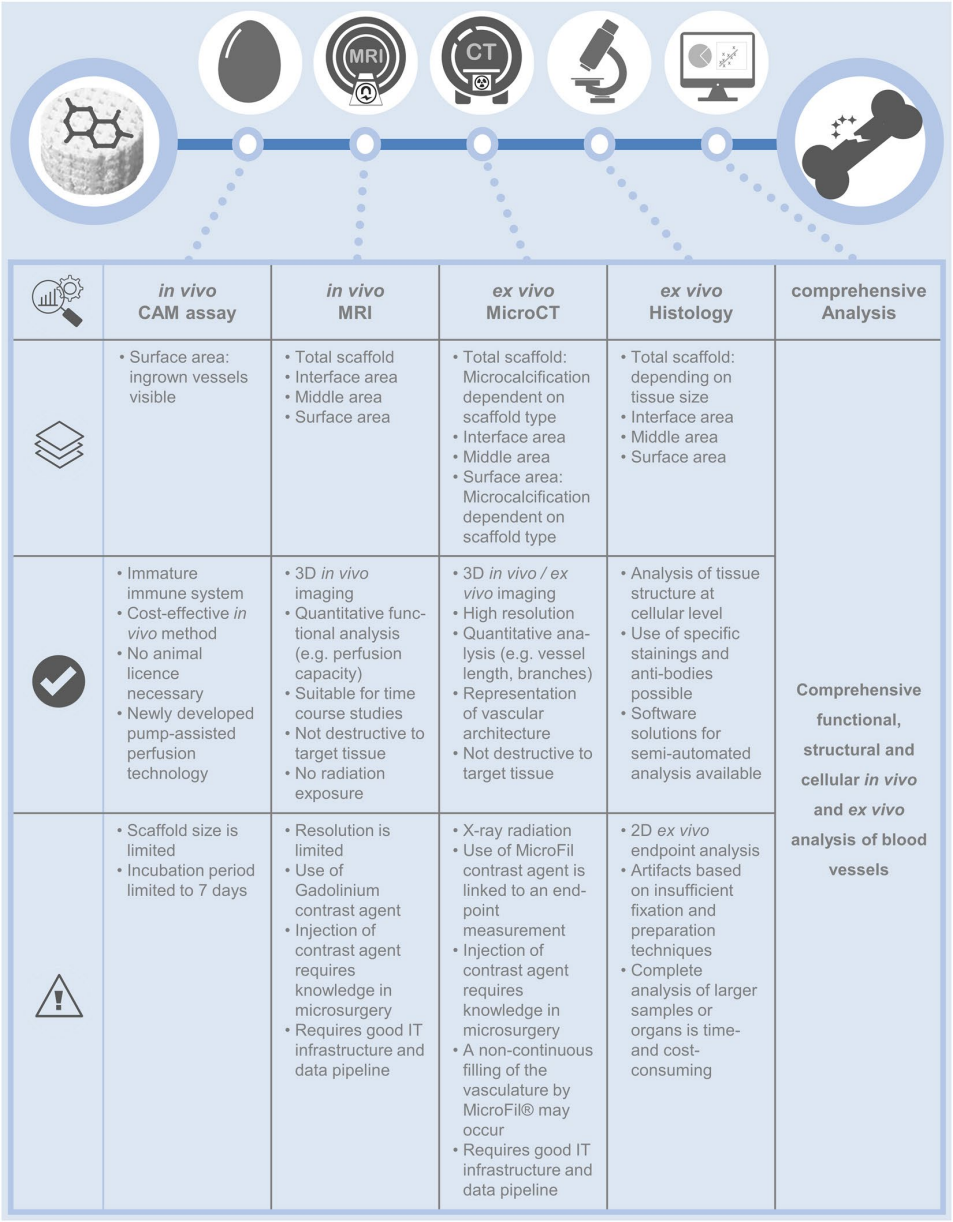
Overview of the comprehensive analysis strategy for the evaluation of biomaterials in bone regenerative therapy. The natural or synthetic biomaterial is incubated on the CAM of the chicken egg. The neo-vascularization of the scaffold is examined by MRI for its functionality, by MicroCT for the vascular architecture and by histology for the vascular pattern. Together, these quantitative results provide a comprehensive assessment of the ingrown blood vessels. The methods used are presented in tabular form and an in-depth evaluation of the individual layers of the scaffold as well as the advantages and technical limitations of each method are listed.

Besides the CAM assay being time- and cost-effective, the two imaging techniques used in our study to visualize neoangiogenesis in biomaterials are complementary and of high clinical relevance. The MRI represents an optimal tool for *in vivo* monitoring of the newly formed functional blood vessels within the scaffold after implantation due to its non-invasive and radiation-free properties. In contrast, the MicroCT works with the help of a micro-focus x-ray source at a very high resolution. After vascular corrosion casting, digital cross-sections of the blood vessel network can be recorded and used to recreate a virtual 3D model without destroying the original object. Furthermore, corrosion casting does not interfere with subsequent histological analyses if needed. Taken together, the analysis of the collected data allows for a comprehensive characterization and direct comparison of functional and structural parameters of blood vessel networks grown inside biomaterials cultured on the CAM. Strengths as well as potential challenges of our comprehensive approach are summarized in Fig. 8.

Our newly developed pump-assisted perfusion technology for the CAM model allows for a more even, reliable and efficient distribution of MicroFil in the vessel system compared to the conventional manual injection^36^, resulting in a consistent quality of contrast medium injection. An extensive literature search identified only a few other studies using a similar combination of MRI and MicroCT analyses to assess vascular function and structure. These studies were limited to sheep^25^ and rodent animal models, i.e. mice^24,47–49^ and rabbits^27^, and were all focusing on either bone TEC vascularization or the analysis of tumour angiogenesis, which is another highly relevant application field for our multimodal imaging approach. Correlation coefficients between those studies and our study were comparable.

### Considerations and potential implications for future bone regeneration strategies

The collagen-based Optimaix scaffold was originally designed for bone tissue engineering (www.matricel.net/de/produkte/optimaix.html). As collagen fibrils are naturally highly mineralized in bone^50,51^, the collagen in Optimaix supported deposition of calcium phases^52^ when placed on the CAM *in ovo*. Control experiments with Optimaix in water, phosphate buffered solution (PBS) or culture medium did not show any such depositions when analysed by MicroCT (data not shown). It was the unique microenvironment encountered on the CAM surface that triggered the microcalcification in Optimaix. In contrast, DegraPol as a synthetic polymer showed only minor calcifications, although - such as Optimaix - it was originally fabricated to serve as a bone substitute^31^.

Finally, for the development of clinically relevant TECs for bone regeneration, a comprehensive analysis of the degree of neovascularization is necessary in addition to its mechanical strength. Important factors such as the nature of the biomaterial, topography, pore size and structure, biocompatibility, as well as cell migration and tissue integration potential are crucial to the use of a biomaterial as a future implant. Sufficient vascularization contributes to nutrient supply, cell metabolism and tissue function. Our study shows for the first time that the use of the CAM assay, together with our novel imaging approach, enables for the comprehensive and standardized monitoring and quantification of neoangiogenesis in 3D format. Hence, valuable information about the regenerative capacity can be obtained for a large variety of potential biomaterial-based implants which represents important data for the safe and efficient translation of such regenerative implants from bench to beside.

### Limitations

First, it needs to be mentioned that the established semi-automatic perfusion of the CAM does not fully correspond to the perfusion technique used in lager mammals. Second, since the heart of the chicken embryo is not accessible for perfusion without damaging the CAM and thus the blood vessels it contains, perfusion is initiated via a vessel on the surface of the CAM. In this case, the blood vessels themselves are perfused not by exchanging MicroFil with blood in a closed circulation, but it is based on the principle of displacing the blood from the blood vessels. The displacement of the blood causes the MicroFil to accumulate towards the end of the blood vessels. An increasing mean radius was measured in the interface towards the surface zone, indicating a lack of circulation of the MicroFil during the perfusion process. However, this did not reveal any disadvantages in the presentation of the vascular architecture by MicroCT measurement. Similar to studies performed in mice and rats, heterogenous perfusion was observed especially in smaller blood vessels, which has been described as one of the major limitations of MicroFil^53–56^. Another limitation is the resolution of the MicroCT scanner. As ultra-high resolution MicroCT machines will become more and more available, the resolution should be chosen to be as high as possible to visualize smaller blood vessels and capillaries.

Finally, the degree of microcalcification of the corresponding scaffold material *in vivo* depends on the composition of the biomaterial. This must be taken into account when analysing MicroCT data, as strong microcalcification can lead to false positive blood vessels in the computer-assisted analysis. However, for the MRI data this artefact has no effect on the result. Hence, depending on the specific scaffold, a despeckling step may have to be considered.

## Conclusions

Here, we present a new, combined MRI/MicroCT analysis for the profound morphological and functional characterization of neovascularization of biomaterials using the CAM *in ovo* model system. To the best of our knowledge, this is the first description of such an analysis. Our novel approach represents an important tool to pre-screen a wide range of biomaterials and TEC for their angiogenic potential and characteristics in a time- and cost-effective, easy and highly reproducible manner. Furthermore, it also helps to replace, reduce, and refine the use of animal models (3 R rule). Therefore, our new approach makes the CAM assay an attractive model system that may bridge the translational gap between experimental *in vitro* data and animal based *in vivo* data to efficiently study neoangiogenesis and even tumour angiogenesis. Our study further highlighted how initial scaffold structure influences and determines the final vascularization pattern, which may be highly relevant for the potential efficacy or failure of a biomaterial in bone regeneration strategies.

## Methods

### Scaffolds

For the corresponding application for bone regeneration (Fig. 1), relevant natural and synthetic biomaterials were investigated for their neo-angiogenesis capacity. The feasibility of evaluating the two biomaterials through comprehensive analysis was demonstrated, considering the different structures of the scaffold for the vessel ingrowth and the different degrees of microcalcification *in vivo*.

Optimaix (Matricel GmbH, Herzogenrath, Germany) is a natural biodegradable material produced from porcine collagen type I. The 3D macroporous scaffold has highly oriented pores of 80–100 µm, which provide a guiding structure for ingrowing cells and blood vessels. The stiffness of the dry Optimaix scaffold was determined to be 148 kPa, while the stiffness of the wet Optimaix scaffold decreased to 7.5 kPa^57^.

DegraPol is a synthetic biodegradable and highly porous polyester-urethane. It is a block co-polymer that consists of polyhydroxy-butyrate and ε-caprolactone^58^. Foams were kindly provided by Ab Medica SpA (Medolla, Italy). After being soaked in cyclohexane and overnight freezing at −20 °C they were cut and dried at room temperature and sterilized with ethylene oxide. The pore size ranged between 300–400 µm. A previous study described DegraPol to have a storage modulus of 105 kPa after 2 weeks of static culture^59^.

Prior to use, both sterile packed Optimaix and sterilized DegraPol scaffolds were cut into equally sized cylinders of 5 mm in diameter and 3 mm height.

### CAM assay

For experiments in chicken embryos until embryonic day 14 no IACUC approval is required according to Swiss animal care guidelines (TSchV, Art. 112). Briefly, fertilized Lohman white LSL (Lohmann Selected Leghorn) chicken eggs (Animalco AG, Staufen AG, Switzerland) were incubated at 37 °C and 65% humidity for 3 days. Using a drill, a window was created in the eggshell after removing 4 mL of albumen. The window was covered with a Petri dish and incubated at 37 °C for another 4 days as described earlier^9^. On incubation day (ID) 7, scaffolds were immersed in sterile phosphate buffered saline (PBS). For stabilization, two scaffolds per egg were placed in the middle of silicone rings (outer diameter: 12 mm) on the CAM of the chicken eggs, which were incubated for 7 more days. The ingrowth of vessels into the biomaterials was observed and documented daily using a stereoscope (Leica EZ4 HD, Leica Microsystems AG, Heerbrugg SG, Switzerland)^14^.

### Magnetic resonance imaging (MRI)

On ID 14, MRI was performed with a 4.7 T/16 cm Bruker PharmaScan small animal scanner (Bruker BioSpin MRI GmbH, Ettlingen, Germany)^9^. Briefly, eggs were sedated with 0.3 mL of 0.3 mg/kg medetomidine (Dorbene ad us. vet., Injektionslösung, Dr. E. Graeub AG, Bern BE, Switzerland)^60^. T_1_-weighted MR images were acquired with a RARE sequence of variable TR and TE for quantitative T_1_ and T_2_ mapping (TR 200/400/800/1500/3000/4500 ms, TE 10/30/50/70/90 ms, RARE-factor 2, image matrix size 220 × 150, field of view 45 × 30 mm^2^, spatial resolution 0.2 × 0.2 mm^2^, slice thickness 1 mm, total scan time 9 min 40 s). Before and 25 minutes after i.v. injection of 100 µL 0.05 M Gd-DOTA MRI contrast agent (Dotarem, Guerbet AG, Zürich ZH, Switzerland) T_1_ maps were acquired. T_1_ relaxation times were determined in three regions of interest (interface, middle and surface). The height of the scaffold was divided into three sections of equal thickness for this purpose. The bottom part is defined as the interface layer, which is in contact with the CAM, followed by the middle layer, and the surface layer, which is exposed to air (Fig. 3b). Changes in the longitudinal relaxation rate, ∆R_1_ = R_1_-R_10_ (with R_1_ = 1/T_1_ and R_1_ = R_10_ + r_1_ [Gd] and r_1_ = relaxivity), before and after injection of Gd-DOTA, were attributed to perfusion capacity of functional vessels in the scaffolds.

### MicroFil perfusion

A new technique for semi-automated perfusion of the contrast agent was established and adapted to the CAM of the chicken egg (Fig. 2). In contrast to manual approaches, our new semi-automated technique provides a higher perfusion efficiency with a homogenous perfusion of most of the vascular network of the chicken embryo. In addition, also the perfusion pressure can be controlled thereby avoiding vessel dilatation and leakage. Right after the MRI (Fig. 2h), and in the case of the time course experiment without previous MRI, eggs were prepared for the perfusion with MicroFil (Flow Tech, Inc., Carver, MA, USA) by stabilizing the shell edges with tape before cutting down the eggshell to the level of the CAM. The freshly mixed MicroFil consisted of 1 mL silicone rubber injection compound (yellow), which was diluted in 8 mL of MV-Diluent and activated by the addition of 1 mL MV-Curing Agent right before use resulting in a working time of at least 20 min. For efficient perfusion, the MicroFil was pumped through a tube with an attached injection needle (30 G ½″) at 0.1 mL/min after the tube has been washed in a 0.9% sodium chloride (NaCl) solution containing 20 i.E./mL of heparin (Bichsel AG, Interlaken, Switzerland; Fig. 2a,b). The pump was stopped when MicroFil had reached the tip of the injection needle. The silicone rings were removed, and the needle was carefully inserted at a branching point into one of the larger blood vessels that were attached to the CAM (Fig. 2c–e). For better resistance, the CAM was held with blunt end forceps at a distance of max. 1 cm behind the injection point. If the CAM was too dry and visibility of the vessels reduced, a few drops of NaCl solution were used in the injection area. Once the needle was inside the vessel, the pump was started by a second investigator who was assisting during the perfusion (Supplementary Video S1). If leakage occurred or vessels in and/or around the scaffolds remained non-perfused, the pump was stopped immediately. Excessive MicroFil was absorbed with the help of cotton swabs and gauze. The needle was carefully removed from the vessel and was replaced by a new one before other large vessels with easy access points were targeted and perfusion was continued. After sufficient perfusion, the eggs were kept at 4 °C overnight to allow for polymerization of MicroFil. In the morning 3 mL of 4% formalin (Kantonsapotheke, Zurich, Switzerland) were injected under the CAM and another 1–2 mL were used to cover the samples and the top of the CAM. Finally, 4 h later the samples were excised using blunt end forceps and small pointy scissors before they were placed in wells of a 24-well-plate filled with 1 mL of 4% formalin and incubated at 4 °C overnight. The samples were washed in PBS and stored in 70% ethanol at 4 °C until the MicroCT scan was performed. MicroFil perfusion was not limited to the superficial vessels of the CAM, but filled the vasculature systemically, as can be seen in the MicroCT image of the chicken embryo head (Fig. 2f,g). Especially the coronal view of the chicken embryo head clearly shows that even the major circulus arteriosus of the iris were perfused with MicroFil (white arrow), which confirms a successful and complete perfusion technique.

### Microcomputed tomography

MicroFil-perfused biomaterials were carefully wrapped in cling foil without squishing before MicroCT scanning was performed on a Skyscan 1176 *in vivo* MicroCT device (Bruker BioSpin AG, Fällanden ZH, Switzerland). The samples were scanned at an isotropic nominal resolution of 18 µm, at 50 kV, 500 µA, with an exposure of 280 ms, and two-fold frame averaging. The collected data was processed using software provided by the manufacturer and ImageJ v1.51k (National Institutes of Health, Rockville MD, USA). Quantitative analysis of the vessel architecture was performed using the ImageJ macro for blood vessel segmentation and network analysis provided by the Advanced Digital Microscopy Core Facility at the Institute for Research in Biomedicine (Barcelona, Spain)^61^. The vessel volume, the number of branches, junctions, branches per junctions, total and mean branch lengths, as well as the mean radius were determined.

### Histological analysis

The scaffolds were embedded in paraffin after MicroCT measurement and sectioned at a thickness of 5 μm. Tissue sections were stained with Haematoxylin (Artechemis AG, Zofingen AG, Switzerland) and Eosin (Waldeck GmbH & Co. KG, Münster, Germany; H&E). Images were taken with a digital slide-scanner NanoZoomer using NDP.view2 software (Hamamatsu Photonics, Japan). The vessel density of each sample was assessed at a magnification of 40x. Both the blood vessels filled with MicroFil and red blood cells were included in the quantitative analysis. The region of interest covered the entire area of the scaffold per section and the results were expressed per mm^2^ of scaffold area in each section. The vessel directions were measured by aligning the scaffold sections horizontally and dividing the long axis of each vessel by the short axis of each vessel. Only vessels with a ratio equal or higher than 3 were taken into account to ensure that only vessels that were sectioned sagitally would contribute to this measurement.

### Statistics

Comparisons of the groups were performed using one-way analysis of variance (ANOVA). When there were significant differences, comparisons between the groups were further assessed with Bonferroni multiple-comparison test. Data were considered statistically significant at *p* < 0.05 and highly significant at *p* < 0.01.

## Supplementary information

Supplementary Information

Supplementary Video S1

## Data Availability

All raw and processed image data generated during and/or analysed during the current study are available from the corresponding author on reasonable request.
