# Supplementary material for: Novel multimodal MRI and MicroCT imaging approach to quantify angiogenesis and 3D vascular architecture of biomaterials
Source: Sci Rep. 2019 Dec 19;9:19474. doi: 10.1038/s41598-019-55411-4 (PMC6923434; doi:10.1038/s41598-019-55411-4)
Supplement: Supplementary file 1 — Supplementary Information [file 41598_2019_55411_MOESM1_ESM.docx]

**SUPPLEMENTARY INFORMATION**

**Novel multimodal MRI and MicroCT imaging approach to quantify angiogenesis and 3D vascular architecture of biomaterials**

Anna Woloszyk, Petra Wolint, Anton S. Becker, Andreas Boss, Weston Fath, Yinghua Tian, Simon P. Hoerstrup, Johanna Buschmann and Maximilian Y. Emmert


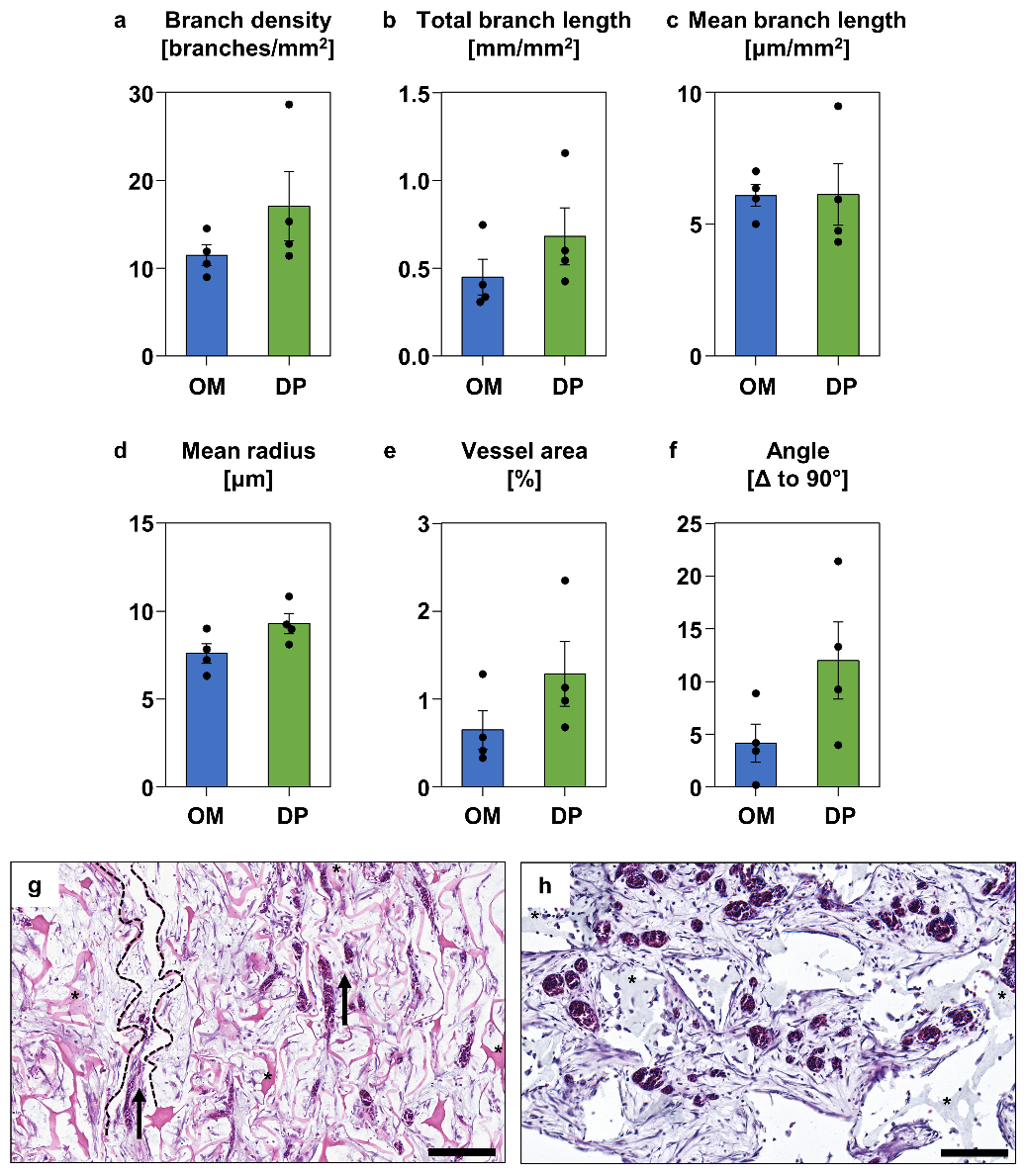


**Figure S1. Histological analysis of H&E-stained sections of Optimaix and DegraPol samples.** **a**, Number of vessels per mm^2^ of scaffold area. **b**, Total branch length in mm per mm^2^ of scaffold area. **c**, Mean branch length in mm per mm^2^ of scaffold area. **d**, Mean radius in μm. **e**, Vessel area in %. **f**, Angle difference from 90°. **g**, H&E staining of an Optimaix scaffold. Asterisks indicate collagen fibers of the scaffold. Dashed line highlights a pore within the scaffold. Black arrows show the direction of the ingrowing blood vessels. **h**, H&E staining of a DegraPol scaffold. Asterisks indicate the scaffold material. Abbreviations: DP - DegraPol, OM - Optimaix. All measurements were taken from distinct samples. Mean ± SE, n = 4, scale bar = 200 μm.

**Table S1.** **Correlation coefficients R for the comparison of MicroCT readouts with perfusion capacity assessed by MRI for the interface, middle, and surface.** Key: white -0.25<R<0.25 (no correlation); light grey -0.5<R<-0.25 & 0.25<R<0.5 (weak correlation); medium grey -0.75<R<-0.5 & 0.5<R<0.75 (moderate correlation); dark grey -1<R<-0.75 & 0.75<R<1 (strong correlation).

|  |  | **Optimaix** | | **DegraPol** | |
| --- | --- | --- | --- | --- | --- |
|  | **MRI vs. MicroCT parameters** | **before despeckle** | **after**  **despeckle** | **before despeckle** | **after despeckle** |
| **INTERFACE** | Total vessel volume (mm^3^) | 0.3854 | 0.4187 | 0.1142 | -0.2418 |
|  | # Branches | 0.7585 | 0.2541 | -0.6152 | 0.378 |
|  | # Junctions | 0.6975 | 0.5827 | -0.602 | -0.3631 |
|  | # Branches/Junction | -0.752 | -0.5934 | 0.3878 | 0.5933 |
|  | Total branch length (mm) | 0.7384 | 0.601 | 0.05386 | -0.1565 |
|  | Mean branch length (mm) | 0.4959 | 0.47 | 0.9479 | -0.5765 |
|  | Mean vessel radius (mm) | -0.7879 | -0.6766 | 0.6114 | -0.03088 |
| **MIDDLE** | Total vessel volume (mm^3^) | 0.09595 | 0.8414 | 0.9094 | -0.574 |
|  | # Branches | -0.1859 | 0.8128 | 0.07018 | -0.8033 |
|  | # Junctions | -0.5539 | 0.5221 | 0.02697 | -0.7783 |
|  | # Branches/Junction | 0.8876 | 0.8173 | -0.2345 | -0.03226 |
|  | Total branch length (mm) | -0.4538 | 0.9724 | 0.3066 | -0.7749 |
|  | Mean branch length (mm) | -0.08257 | -0.805 | 0.3716 | 0.911 |
|  | Mean vessel radius (mm) | 0.002442 | 0.562 | 0.7922 | 0.8467 |
| **SURFACE** | Total vessel volume (mm^3^) | 0.7551 | 0.9276 | -0.2136 | -0.6925 |
|  | # Branches | -0.6577 | 0.9276 | -0.3181 | 0.8947 |
|  | # Junctions | -0.6719 | 0.5319 | -0.2887 | 0.8798 |
|  | # Branches/Junction | -0.3863 | -0.1897 | 0.4522 | 0.1122 |
|  | Total branch length (mm) | 0.6456 | 0.8298 | -0.2426 | 0.8203 |
|  | Mean branch length (mm) | 0.6388 | -0.8854 | 0.8476 | -0.4949 |
|  | Mean vessel radius (mm) | 0.6348 | 0.832 | -0.1624 | 0.01373 |

**Video S1:** **MicroFil perfusion of the CAM.**
